# Effects of overweight and the *PLA2G7* V279F polymorphism on the association of age with systolic blood pressure

**DOI:** 10.1371/journal.pone.0173611

**Published:** 2017-03-23

**Authors:** Minjoo Kim, Minkyung Kim, Hye Jin Yoo, Hye Young Jang, Sang-Hyun Lee, Jong Ho Lee

**Affiliations:** 1 Research Center for Silver Science, Institute of Symbiotic Life-TECH, Yonsei University, Seoul, Korea; 2 National Leading Research Laboratory of Clinical Nutrigenetics/Nutrigenomics, Department of Food and Nutrition, College of Human Ecology, Yonsei University, Seoul, Korea; 3 Department of Food and Nutrition, Brain Korea 21 PLUS Project, College of Human Ecology, Yonsei University, Seoul, Korea; 4 Department of Family Practice, National Health Insurance Corporation, Ilsan Hospital, Goyang, Korea; University of Insubria, ITALY

## Abstract

This prospective study aimed to determine the effects of the persistence of overweight for three years and the *PLA2G7* V279F polymorphism, as well as the interaction between these factors, on the association of age with blood pressure (BP). Healthy middle-aged subjects with normotensive BP were divided into the normal-weight and overweight groups. The *PLA2G7* V279F genotype, BP, lipoprotein-associated phospholipase A_2_ (Lp-PLA_2_) activity, and oxidized low-density lipoprotein (ox-LDL) were determined. Lp-PLA_2_ activity was lower in the F allele subjects (*n* = 111) than in those with the VV genotype (*n* = 389). The overweight individuals with the F allele had lower Lp-PLA_2_ activity and ox-LDL at both baseline and after three years and lower systolic and diastolic BP and LDL cholesterol after three years compared with those with the VV phenotype. After three years, the overweight subjects with the VV phenotype exhibited greater increases in Lp-PLA_2_ activity, systolic BP, and ox-LDL than those with the F allele and normal-weight subjects with the VV phenotype. A multivariate analysis revealed that the *PLA2G7* V279F genotype, baseline BMI, changes in Lp-PLA_2_ activity and ox-LDL remained independently and positively associated with changes in systolic BP. The simultaneous presence of the *PLA2G7* 279VV genotype and persistence of overweight synergistically increases the risk for hypertension, whereas lower Lp-PLA_2_ activity in *PLA2G7* 279F allele carriers might offer certain protection against hypertension, even in individuals who have been overweight for over three years.

## Introduction

The single nucleotide polymorphism phospholipase A2 group VII (*PLA2G7*) V279F, a missense mutation in the *PLA2G7* gene, is found in approximately 12% of the Korean population (0.5-2% homozygosity) [1–3]. The 279F allele protects against coronary artery disease (CAD) in Korean men [1]. Several studies conducted in Korea [2–4] have demonstrated that homozygous carriers of this variant lack the enzyme in plasma and that heterozygous carriers have approximately 60-75% of the activity detected in individuals carrying two copies of the wild-type allele. A direct correlation of lipoprotein-associated phospholipase A_2_ (Lp-PLA_2_) with blood pressure (BP) was recently reported [5]; however, the causative role of Lp-PLA_2_ in hypertension is unknown.

A prospective study with *PLA2G7* 279 F allele subjects provides a natural experiment to gain insight into the causal contribution of Lp-PLA_2_ to the pathogenesis of age-related hypertension. Therefore, the objective of this prospective study was to determine the effects of the persistence of overweight during the three-year study period and the genetic variants of *PLA2G7* V279F, as well as the interaction between these factors, on the association of age with BP in healthy middle-aged subjects with normotensive BP.

## Materials and methods

### Study population

The study participants were recruited from a three-year prospective cohort study including 800 healthy subjects at the health-promotion center of Ilsan Hospital during routine checkup visits between January 2007 and May 2011. Based on the data obtained from the health-promotion center, the subjects who met the study criteria and agreed to participate were referred to the Department of Family Medicine. The potential subjects’ health, including BP, was reassessed, and the subjects who met the study criteria (systolic BP < 140 mmHg and diastolic BP < 90 mmHg) were then recommended to participate. A total of 500 participants aged 35-60 years were ultimately selected according to the study criteria. Clinical and blood tests, including BP measurements, were performed again at the baseline visit. The exclusion criteria were hypertension (systolic BP 140 mmHg or diastolic BP ≥ 90 mmHg, or current use of antihypertensive medication); current and/or history of cardiovascular disease, diabetes mellitus, dyslipidemia, liver disease, renal disease, pancreatitis, or cancer; pregnancy or lactation; and regular use of any medication. The aim of the study was carefully explained to all of the participants, who provided written informed consent. The Institutional Review Board of Yonsei University and Ilsan Hospital approved the study protocol, which complied with the Declaration of Helsinki.

### Genotyping of *PLA2G7* V279F

Genomic DNA was extracted from 5 mL of whole blood using a commercially available DNA isolation kit (WIZARD® Genomic DNA purification kit, Promega Corp., Madison, WI, USA) according to the manufacturer’s recommended protocol. V279F (rs76863441) genotyping was performed through a single-base primer extension assay using the SNaPShot assay kit (Applied Biosystems Inc., Foster City, CA, USA) according to the manufacturer’s recommended protocol.

### BP and brachial-ankle pulse wave velocity

The BP was measured using a random-zero sphygmomanometer (HM-1101, Hico Medical Co., Ltd., Chiba, Japan) with appropriately sized cuffs after at least a 20-minute rest period in the sitting position. BP readings were obtained from both arms, and the higher of the two readings was recorded. Three BP measurements were obtained at each visit, and the differences between the three systolic BP readings were always less than 2 mmHg. The average value of the readings was used as a measure of the systolic and diastolic BP values. The participants were instructed not to smoke or drink alcohol for at least 30 minutes before each BP measurement. The brachial-ankle pulse wave velocity (baPWV) was measured using an automatic waveform analyzer (model VP-1000; Nippon Colin Ltd., Komaki, Japan) as previously described [6].

### Clinical and biochemical assessments

Detailed information on the clinical and biochemical assessments is provided elsewhere [7]. The body weight, height, and waist circumference were measured, and the BMI was calculated in units of kilograms per square meter (kg/m^2^). Blood samples were collected following an overnight fast of at least 12 hours. The levels of fasting triglycerides, total high-density lipoprotein (HDL) cholesterol, low-density lipoprotein (LDL) cholesterol, glucose, insulin, and oxidized LDL (ox-LDL) were measured as previously described [7]. Insulin resistance (IR) was determined by the homeostasis model assessment (HOMA) using the following equation: HOMA-IR = [fasting insulin (μIU/mL) × fasting glucose (mmol/L)] / 22.5. The level of 8-epi-prostaglandin (PG) F_2α_ was measured using a Urinary Isoprostane ELISA kit (Oxford Biomedical Research Inc., Rochester Hills, MI, USA).

### Estimation of sodium intake

The participants completed a semi-quantitative food frequency questionnaire and a 24-h recall with the assistance of a dietitian at baseline. A computerized version of the Korean Nutrition File (Can-Pro 3.0; The Korean Nutrition Society, Seoul, Korea) was used to determine the sodium intake.

### Statistical analysis

Statistical analyses were performed using SPSS version 21.0 (IBM/SPSS, Chicago, IL, USA). Hardy-Weinberg equilibrium was assessed using PLINK version 1.07 (http://pngu.mgh.harvard.edu/purcell/plink/). The differences in clinical variables between the two groups (normal-weight vs. overweight groups; VV vs. F allele) were tested by an independent *t*-test. Paired *t*-tests were performed to determine the differences between the baseline and three-year follow-up values for each group. The interactions between genotype and body weight were tested through two-way analysis of variance. Multiple linear regression analyses using the enter method were performed to identify major independent predictors of changes in the systolic and diastolic BP values. Pearson’s correlation coefficient was used to examine the relationships between variables. Heat maps were created to visualize and evaluate the relationships between metabolites and the biochemical measurements in the study population. Logarithmic transformations were performed for skewed variables. The results are expressed as the means ± standard errors (SEs), and a two-tailed *P*-value < 0.05 was considered statistically significant.

## Results

### Frequency of the *PLA2G7* V279F polymorphism in normal-weight and overweight subjects

We divided the cohort into two groups: normal weight (18.5 kg/m^2^ ≤ BMI < 25 kg/m^2^, *n* = 352) and overweight (25 kg/m^2^ ≤ BMI < 30 kg/m^2^, *n* = 148). The *PLA2G7* V279F genotype distribution among the 352 normal-weight subjects was as follows: 278 subjects were homozygous for the V allele (VV), 69 were heterozygous for the F allele (VF), and five were homozygous for the F allele (FF). The distribution of the *PLA2G7* V279F genotype among the 148 overweight subjects was the following: 111 had the VV genotype, and 37 had the VF genotype. These frequencies did not deviate significantly from Hardy-Weinberg equilibrium (*P* > 0.05). The minor allele frequencies were 0.112 and 0.125 in the normal-weight and overweight individuals, respectively, which is consistent with our previous observations in Korean subjects [1–3]. We pooled the heterozygotes (VF) and rare allele homozygotes (FF) to increase the statistical power.

### Clinical characteristics and biochemical parameters according to the *PLA2G7* V279F genotype at baseline and at the end of the three-year follow-up

With regard to the *PLA2G7* V279F polymorphism, there were no significant differences in the baseline age and gender distributions across genotypes between the normal-weight and overweight groups (Table 1). Similarly, no significant differences in smoking and drinking status across genotypes were detected between the normal-weight and overweight groups at baseline and at the end of the three-year follow-up (data not shown).

**Table 1:** Association of *PLA2G7* V279F genotypes with clinical and biochemical characteristics at baseline and at the end of the three-year follow-up according to BMI.

	Normal weight (n = 352)				Overweight (n = 148)			
	VV (n = 278)		F allele (n = 74)		VV (n = 111)		F allele (n = 37)	
	Baseline	Follow-up	Baseline	Follow-up	Baseline	Follow-up	Baseline	Follow-up
Age (year)	47.9±0.44		47.5±0.91		47.5±0.75		48.9±1.29	
Male (n (%))/Female (n (%))	131 (47.1) / 147 (52.9)		30 (40.5) / 44 (59.5)		53 (47.7) / 58 (52.3)		17 (45.9) / 20 (54.1)	
BMI (kg/m^2^)	22.1±0.11	22.2±0.12	21.9±0.22	21.9±0.23	26.6±0.14^*c*^	26.6±0.16^*d*^	26.8±0.35^*e*^	26.8±0.43^*f*^
Waist (cm)	80.1±0.37	81.7±0.42***	80.7±0.61	82.1±0.76*	88.2±0.59^*c*^	90.4±0.55^*d*^ ^,^**	91.1±0.87^*a*^^,^^*e*^	90.7±0.98^*f*^
Diastolic BP (mmHg)	71.2±0.63	72.0±0.65	70.8±1.29	72.1±1.18	76.2±0.86^*c*^	81.0±1.16^*d*^^,^***	75.3±1.73^*e*^	75.7±1.53^*b*^
Change	0.77±0.62		1.38±1.24		4.84±0.91^*i*^		0.46±1.69	
Triglyceride (mg/dL)^*∮*^	99.3±3.71	106.6±4.56	89.1±5.78	88.1±5.72	135.8±7.36^*c*^	138.1±7.89^*d*^	156.3±14.9^*e*^	166.1±16.9^*f*^
Total-cholesterol (mg/dL)^*∮*^	188.1±2.00	198.7±2.31***	190.7±3.75	191.8±3.24	194.7±3.13	208.7±3.89^*d*^^,^***	199.0±4.55	194.5±4.49
Change	10.6±1.94		1.11±3.36^*g*^		14.1±3.05		-4.49±4.10^*h*^	
HDL-cholesterol (mg/dL)^*∮*^	54.7±0.90	52.3±0.79**	57.2±1.60	54.7±1.57*	49.8±1.23^*c*^	45.7±1.32^*d*^^,^***	50.0±1.80^*e*^	48.1±1.61^*f*^
LDL-cholesterol (mg/dL)^*∮*^	114.0±1.93	126.0±2.11***	115.7±3.53	119.5±3.17	117.9±2.91	135.6±3.36^*d*^^,^***	118.3±5.00	113.1±4.70^*b*^
Change	12.0±1.93		3.77±3.71		17.7±2.97		-5.17±4.50^*h*^	
Glucose (mg/dL)^*∮*^	90.0±0.53	91.6±0.53**	90.0±1.11	90.5±1.09	95.0±1.04^*c*^	96.5±1.24^*d*^	97.0±1.60^*e*^	98.3±1.85^*f*^
Insulin (μIU/dL)^*∮*^	7.97±0.18	7.36±0.18**	7.98±0.37	7.31±0.35	9.82±0.37^*c*^	9.01±0.37^*d*^ ^,^*	9.91±0.65^*e*^	9.89±0.84^*f*^
HOMA-IR^*∮*^	1.78±0.04	1.67±0.04*	1.78±0.09	1.64±0.08	2.32±0.10^*c*^	2.18±0.10^*d*^	2.36±0.18^*e*^	2.39±0.24^*f*^
8-epi-PGF_2α_ (pg/mg creatinine)^*∮*^	1382.0±36.1	1424.0±34.6	1380.6±60.1	1401.4±61.8	1409.3±50.1	1490.8±43.0^*d*^	1412.9±134.9	1354.5±77.6
baPWV (cm/s)^*∮*^	1296.4±11.3	1310.5±12.1	1270.8±19.5	1289.7±24.9	1325.6±17.2	1360.1±19.6^*d*^^,^**	1303.1±30.8	1313.3±29.6

Mean ± SE.

^*∮*^tested by logarithmic transformation.

^*a*^*P*<0.05, comparison between individuals with the VV genotype and F allele in the overweight group at baseline.

^*b*^*P*<0.05, comparison between individuals with the VV genotype and F allele in the overweight group at the end of the three-year follow-up.

^*c*^*P*<0.05, comparison of individuals with the VV genotype between the normal-weight and overweight groups at baseline.

^*d*^*P*<0.05, comparison of the individuals with the VV genotype between the normal-weight and overweight groups at the end of the three-year follow-up.

^*e*^*P*<0.05, comparison of the individuals with the F allele between the normal-weight and overweight groups at baseline.

^*f*^*P*<0.05, cmparison of the individuals with the F allele between the normal-weight and overweight groups at the end of the three-year follow-up.

^*g*^*P*<0.05, comparison between individuals with the VV genotype and F allele in the normal-weight group.

^*h*^*P*<0.05, comparison between individuals with the VV genotype and F allele in the overweight group.

^*i*^*P*<0.05, comparison of the individuals with the VV phenotype between the normal-weight and overweight groups.

*P<0.05

**P<0.01, and

***P<0.001 compared with the levels at baseline of each group, as determined through a paired t-test.

**Fig 1:**
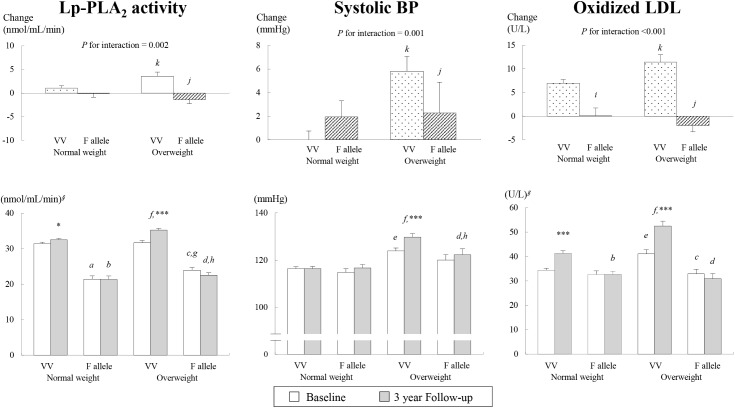
Genotype effect of *PLA2G7* V279F on changes in Lp-PLA_2_ activity, systolic BP, and oxidized LDL in the normal-weight and overweight groups at the end of the three-year follow-up compared with the baseline. Mean ± SE. ^*∮*^tested by logarithmic transformation. ^*a*^*P*<0.05, comparison between the VV genotype and F allele in the normal-weight group at baseline. ^*b*^*P*<0.05, comparison between the VV genotype and F alleles in the normal-weight group at the end of the three-year follow-up. ^*c*^*P*<0.05, comparison between the VV genotype and F allele in the overweight group at baseline. ^*d*^*P*<0.05, comparison between the VV genotype and F allele in the overweight group at the end of the three-year follow-up. ^*e*^*P*<0.05, comparison of the VV genotype between the normal-weight and overweight groups at baseline. ^*f*^*P*<0.05, comparison of the VV allele between the normal-weight and overweight groups at the end of the three-year follow-up. ^*g*^*P*<0.05, comparison of the F allele between the normal-weight and overweight groups at baseline. ^*h*^*P*<0.05, comparison of the F allele between the normal-weight and overweight groups at the end of the three-year follow-up. ^*i*^*P*<0.05, comparison between the VV genotype and F allele in the normal-weight group at change values. ^*j*^*P*<0.05, comparison between the VV genotype and F allele in the overweight group at change values. ^*k*^*P*<0.05, comparison in the VV genotype between the normal-weight and overweight groups at change values. ^*l*^*P*<0.05, comparison of the F allele between the normal-weight and overweight groups at change values. ^*^P<0.05, ^**^P<0.01, and ^***^P<0.001 compared with the levels at baseline in each group, as determined through a paired t-test.

The normal weight group exhibited a genotype effect of *PLA2G7* V279F at baseline; specifically, lower Lp-PLA_2_ activity was observed in the normal-weight individuals with the F allele compared with those with the VV genotype. A genotype effect in Lp-PLA_2_ activity and plasma ox-LDL was observed at the end of the three-year follow-up; specifically, lower Lp-PLA_2_ activity and lower ox-LDL levels were observed in the normal-weight individuals with the F allele than in those with the VV genotype (Fig 1). After three years, the normal-weight VV individuals showed significant increases in Lp-PLA_2_ activity and levels of ox-LDL (Fig 1), total and LDL cholesterol, and glucose and significant decreases in the HDL cholesterol and insulin levels and the HOMA-IR index compared with the values detected at baseline (Table 1). After three years, the normal-weight F allele individuals exhibited a significant decrease in the HDL cholesterol level compared with the baseline values (Table 1).

After three years, the overweight VV individuals showed significant increases in the systolic BP, Lp-PLA_2_ activity, ox-LDL (Fig 1), waist circumference, diastolic BP, total and LDL cholesterol, and baPWV and significant decreases in the levels of HDL cholesterol and insulin compared with the baseline (Table 1). The overweight group had higher values of the BMI, waist circumference, serum triglyceride, glucose, insulin, and HOMA-IR index and lower HDL cholesterol levels at both baseline and at the end of the three-year follow-up compared with the normal-weight group, regardless of the genotype. At baseline, the overweight F allele individuals presented higher values of waist circumference, diastolic BP and Lp-PLA_2_ activity than the normal-weight F allele individuals. The overweight VV subjects had higher values of waist circumference, systolic and diastolic BP and ox-LDL values than the normal-weight VV subjects at both baseline and at the end of the three-year follow-up. Additionally, after three years, the overweight VV individuals had higher levels of total cholesterol, LDL cholesterol, Lp-PLA_2_ activity, urinary 8-epi-PGF_2α_, and baPWV than the normal-weight VV individuals. At the end of the three-year follow-up, the overweight F allele subjects showed greater waist circumference, systolic BP and Lp-PLA_2_ activity than the normal-weight F allele subjects. Furthermore, the overweight individuals with the F allele showed lower Lp-PLA_2_ activity and ox-LDL at both baseline and at the end of the three-year follow-up and lower systolic (Fig 1) and diastolic BP and LDL cholesterol values at the end of the three-year follow-up than those with the VV genotype (Table 1).

Moreover, there were no significant differences of sodium intake between the VV genotype and F allele (S1 Table). Additionally, no significant differences of sodium intake in each genotype were found between the normal-weight and overweight groups.

### Interaction between the *PLA2G7* V279F genotypes and baseline BMI (normal weight vs. overweight) and its association with the three-year changes in Lp-PLA_2_ activity, systolic BP, and ox-LDL

The genotype effects of *PLA2G7* V279F on the mean (±SEs) changes in Lp-PLA_2_ activity, systolic BP, and ox-LDL in the normal-weight and overweight groups at the end of the three-year follow-up are shown in Fig 1. At the end of the three-year follow-up, after adjustment for age, sex, smoking, and drinking, the results showed significant interactions between the *PLA2G7* V279F genotype and baseline BMI associated with changes in Lp-PLA_2_ activity (*P*-interaction = 0.002), systolic BP (*P*-interaction = 0.001), and ox-LDL (*P*-interaction < 0.001). The overweight subjects with the VV genotype exhibited greater increases in Lp-PLA_2_ activity, systolic BP, and ox-LDL compared with those with the F allele and the normal-weight subjects with the VV genotype. Additionally, the normal-weight subjects with the VV genotype presented greater increases in ox-LDL than those with the F allele (Fig 1).

Similarly, significant interactions were found between the *PLA2G7* V279F genotype and baseline BMI associated with changes in diastolic BP (*P*-interaction = 0.004), total cholesterol (*P*-interaction = 0.002), and LDL cholesterol (*P*-interaction < 0.001). The increases in diastolic BP were greater in the overweight VV subjects than in the normal-weight VV subjects (Table 1). The overweight subjects with the VV genotype showed greater increases in the total and LDL cholesterol levels that those with the F allele.

### Correlations of the changes in systolic and diastolic BP values

**Fig 2:**
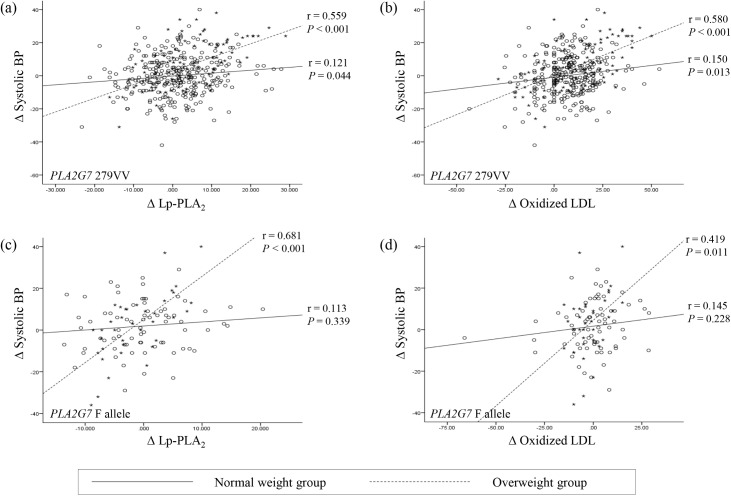
Correlation between changes (∆) in Lp-PLA_2_ and systolic BP and between changes in oxidized LDL and systolic BP in normal-weight (○) and overweight (*) groups according to the *PLA2G7* V279F genotype. (a) Correlation between changes in Lp-PLA_2_ and systolic BP in individuals with the VV genotype. (b) Correlation between changes in oxidized LDL and systolic BP in individuals with the VV genotype. (c) Correlation between changes in Lp-PLA_2_ and systolic BP in individuals with the VF+FF genotype. (d) Correlation between changes in oxidized LDL and systolic BP in individuals with the VF+FF genotype.

The changes in systolic BP over the three-year period were associated with baseline BMI (*P* = 0.006), changes in LDL cholesterol (*P* = 0.008), changes in Lp-PLA_2_ activity (*P <* 0.001), and changes in ox-LDL (*P* < 0.001; Table 2). After adjustment for confounding variables, the *PLA2G7* V279F genotype (*P* = 0.043), baseline BMI (*P* = 0.005), changes in Lp-PLA_2_ activity (*P =* 0.039), and changes in ox-LDL (*P* = 0.003) remained independently and positively associated with changes in systolic BP (Table 2). Fig 2 shows the correlations between the changes in Lp-PLA_2_ activity and systolic BP and between the changes in ox-LDL and systolic BP in the normal-weight and overweight groups according to the *PLA2G7* V279F genotype. The correlations between the changes in Lp-PLA_2_ activity and systolic BP and between the changes in ox-LDL and systolic BP in both the VV and F allele individuals who were overweight were stronger than those found for the normal-weight individuals.

**Table 2:** Correlations of the changes (∆) in systolic blood pressure.

∆ Systolic BP (mmHg)	Univariable analysis		Multivariable analysis	
	Beta-coefficient ± SE	*P*-value	Beta-coefficient ± SE	*P*-value
Age (year)	0.078±0.062	0.211	0.078±0.062	0.203
Sex	-0.538±1.151	0.641	0.617±1.145	0.590
*PLA2G7* V279F genotype	0.387±1.386	0.780	2.836±1.398	**0.043**
Baseline BMI (kg/m^2^)	0.570±0.208	**0.006**	0.573±0.205	**0.005**
∆ Triglyceride (mg/dL)	0.014±0.010	0.146	0.015±0.010	0.140
∆ LDL-cholesterol (mg/dL)	0.048±0.018	**0.008**	0.031±0.019	0.102
∆ HDL-cholesterol (mg/dL)	0.002±0.050	0.960	0.046±0.050	0.361
∆ Lp-PLA_2_ activity (nmol/mL/min)	0.427±0.067	**<0.001**	0.200±0.097	**0.039**
∆ Oxidized LDL (U/L)	0.256±0.040	**<0.001**	0.168±0.056	**0.003**

The β-coefficient is the standardized regression coefficient ± SE. ∆: Change at the end of the three-year follow-up from baseline.

Table 3 shows the correlations of the changes in diastolic BP. The changes in diastolic BP over the three-year period were associated with changes in LDL cholesterol (*P <* 0.001), changes in Lp-PLA_2_ activity (*P <* 0.001), and changes in ox-LDL (*P* < 0.001). A trend toward an association between changes in diastolic BP and baseline BMI was found (*P* = 0.072). After adjustment for these variables, greater changes in LDL cholesterol (*P =* 0.036), Lp-PLA_2_ activity (*P =* 0.043), and ox-LDL (*P* = 0.012) remained independently and positively associated with changes in diastolic BP. After adjustment for other covariates, a trend toward an independent association between the changes in diastolic BP and baseline BMI was detected (*P* = 0.068; Table 3).

**Table 3:** Correlations of changes (∆) in diastolic blood pressure.

∆ Diastolic BP (mmHg)	Univariable analysis		Multivariable analysis	
	Beta-coefficient ± SE	*P*-value	Beta-coefficient ± SE	*P*-value
Age (year)	-0.033±0.050	0.513	-0.035±0.050	0.486
Sex	-0.598±0.925	0.518	0.074±0.928	0.937
*PLA2G7* V279F genotype	-0.856±1.109	0.441	1.429±1.134	0.208
Baseline BMI (kg/m^2^)	0.302±0.168	0.072	0.304±0.166	0.068
∆ Triglyceride (mg/dL)	0.006±0.008	0.414	0.010±0.008	0.243
∆ LDL-cholesterol (mg/dL)	0.052±0.014	**<0.001**	0.032±0.015	**0.036**
∆ HDL-cholesterol (mg/dL)	-0.023±0.040	0.558	0.012±0.040	0.768
∆ Lp-PLA_2_ activity (nmol/mL/min)	0.327±0.054	**<0.001**	0.159±0.078	**0.043**
∆ Oxidized LDL (U/L)	0.192±0.032	**<0.001**	0.115±0.046	**0.012**

The β-coefficient is the standardized regression coefficient ± SE. ∆: Change at the end of the three-year follow-up from baseline.

## Discussion

Age, overweight or obesity, and Lp-PLA_2_ have all been reported to be directly associated with hypertension [5,8]; however, the role played by Lp-PLA_2_ in the development of hypertension remains unknown. This prospective study was designed to examine the effects of the persistence of overweight during the three-year study period and the *PLA2G7* V279F genotype, as well as the interaction between these two factors, on the association of age with BP in healthy middle-aged subjects with normotensive BP. The main finding of this study is that the association of age with systolic BP differed depending on the *PLA2G7* V279F genotype (a missense mutation of the *PLA2G7* gene) [1,2], baseline BMI, and the interaction between these factors. In this study, after adjustment for confounding variables, the *PLA2G7* V279F genotype, as well as higher baseline BMI and greater changes in Lp-PLA_2_, remained significantly and independently associated with greater changes in systolic BP. This independently positive relationship could provide evidence of a potential causality between Lp-PLA_2_ activity and systolic hypertension and partially explains the recent cross-sectional observation of a direct correlation between Lp-PLA_2_ expression and systolic BP in young subjects with metabolic syndrome [5].

A single nucleotide polymorphism, V279F, in chromosome 6 of the *PLA2G7* gene (rs76863441) is known to potently influence enzyme activity. Valine 279 is located near the consensus catalytic site of lipase, and the mutant peptide completely lacks enzymatic activity [9,10]. Similar to previous studies conducted in Korea [2–4], this study demonstrates that homozygous carriers of this variant (FF) lack the enzyme in plasma and that heterozygous carriers (VF) have approximately 70% of the activity detected in individuals carrying two copies of the wild-type allele (VV). After three years, the overweight VV individuals showed significant increases in the systolic and diastolic BP values and levels of LDL cholesterol, Lp-PLA_2_, and ox-LDL, whereas the overweight F allele subjects showed no changes in these variables. Thus, the overweight individuals with the F allele showed lower Lp-PLA_2_, ox-LDL, systolic and diastolic BP, and LDL cholesterol values at the end of the three-year follow-up than those with the VV genotype. These interactive effects between *PLA2G7* V279F and baseline BMI indicate that the approximately 32% lower Lp-PLA_2_ activity detected in the *PLA2G7* 279F allele carriers might offer certain protection against hypertension, even in the case of persistent overweight for over three years. Jang et al. [1] also found that a natural deficiency in Lp-PLA_2_ activity due to carriage of the *PLA2G7* 279F allele protects against CAD in Korean men.

Hypertension, age, overweight or obesity, and Lp-PLA_2_ are major determinants of CAD [11–13], and hypertension is considered an inflammatory disease. Lp-PLA_2_ uses ox-LDL as a substrate and produces oxidized fatty acids and lysophosphatidylcholine (lysoPC), a powerful pro-inflammatory and pro-calcifying factor [14]. LysoPC induces the production of reactive oxygen species by inducing the uncoupling of the endothelial nitric oxide synthase (eNOS) [15,16], and this enzyme becomes a superoxide and peroxynitrite producer and thereby contributes to atherogenesis, plaque destabilization, and hypertension [17]. LysoPC can also increase the level of Lp-PLA_2_, which eventually aggravates the degree of inflammation. In addition, prehypertension and hypertension were recently found to be associated with increased Lp-PLA_2_ activity and elevated levels of circulating lysoPCs and ox-LDL, and a positive correlation between lysoPC and BP has also been reported [18,19]. With respect to the potent effects of Lp-PLA_2_ on promoting vascular inflammation, increased Lp-PLA_2_ activity might be associated with hypertension. A meta-analysis of 32 prospective studies revealed that after correction for other cardiovascular risk factors, the risk of developing CAD was increased by 11% for each standard deviation unit increase in Lp-PLA_2_ activity [20]. The amplitude of the association with CAD was comparable to that of systolic BP in these populations [20]. The present study demonstrated that the correlations between changes in Lp-PLA_2_ activity and systolic BP and between changes in ox-LDL and systolic BP in both VV and F allele individuals who were overweight were stronger than those detected in normal-weight individuals. This result suggests that the simultaneous presence of the persistence of overweight and the *PLA2G7* 279VV genotype can provide a synergistic effect on the acceleration of hypertension in healthy middle-aged subjects with normotensive BP.

Ambulatory BP monitoring was not performed in all of the subjects; therefore, the data presented in this manuscript cannot represent an exact BP. However, patients with chronic disease were excluded; thus, the use of a random-zero sphygmomanometer could be considered a generally appropriate measurement approach. no significant differences between the VV genotype and F allele were detected in each group. Additionally, no significant differences of sodium intake in each genotype were found between the normal-weight and overweight groups, in other words, BP and weight status are not affected by sodium intake. However, these data were driven by an estimated rather than the exact sodium intake as 24-h urinary sodium, thus, the exact amount of sodium intake is unknown. This study provides evidence that the association of age with systolic BP differed depending on the subjects’ *PLA2G7* V279F genotype (a missense mutation of the *PLA2G7* gene), baseline BMI, and the interaction between these two factors. The simultaneous presence of the *PLA2G7* 279VV genotype and the persistence of overweight could synergistically increase the risk for hypertension in healthy middle-aged subjects with normotensive BP. However, the 32% lower Lp-PLA_2_ activity detected in *PLA2G7* 279F allele carriers might offer certain protection against hypertension, even in the case of persistent overweight for more than three years. This result might provide evidence indicating a potential causality between Lp-PLA_2_ activity and hypertension and provides an impetus for reducing Lp-PLA_2_ activity or body weight to prevent progression to advanced hypertension, particularly in overweight subjects with the *PLA2G7* 279VV genotype.

## Supporting information

S1 Table Differences of PLA2G7 V279F genotypes with sodium intake according to BMI at baseline. Mean ± SE. Independent t-test was performed to calculate.
(PDF)
