# Supplementary material for: Effects of overweight and the PLA2G7 V279F polymorphism on the association of age with systolic blood pressure
Source: PLoS One. 2017 Mar 23;12(3):e0173611. doi: 10.1371/journal.pone.0173611 (PMC5363925; doi:10.1371/journal.pone.0173611)
Supplement: S1 Table — Mean ± SE. Independent t-test was performed to calculate. (PDF) [file pone.0173611.s001.pdf]

S1 Table. Differences of *PLA2G7* V279F genotypes with sodium intake according to BMI at baseline.

	**Normal weight (*n*=352)**		***P***	**Overweight (*n*=148)**		***P***
	**VV (*n*=278)**	**F allele (*n*=74)**		**VV (*n*=111)**	**F allele (*n*=37)**	
Sodium intake (mg)	4799.5±155.0	4484.6±184.7	0.319	4704.7±166.0	4296.0±252.6	0.206

Mean ± SE. Independent *t*-test was performed to calculate.
